# HPV Vaccination in India: Critical Appraisal

**DOI:** 10.1155/2014/394595

**Published:** 2014-03-11

**Authors:** Aruna Nigam, Pikee Saxena, Anita S. Acharya, Archana Mishra, Swaraj Batra

**Affiliations:** ^1^Department of Obstetrics and Gynaecology, Hamdard Institute of Medical Sciences and Research, New Delhi 110062, India; ^2^Reader's Flat No. 4, Lady Hardinge Medical College Campus, New Delhi 110001, India; ^3^Department of Obstetrics and Gynaecology, Lady Hardinge Medical College, New Delhi 110001, India; ^4^Department of Preventive and Social Medicine, Lady Hardinge Medical College, New Delhi 110001, India

## Abstract

Cervical cancer is the third most common cancer in women worldwide. The role of human papilloma virus (HPV) in the genesis of cervical carcinoma is well documented. The HPV 16 and 18 are found to be most commonly associated with invasive cervical carcinoma. The advent of cervical carcinoma vaccine has advanced the hopes that eradication of cervical carcinoma might be possible in future. The scenario of prevention of cervical carcinoma is completely different in developed and developing countries. The implementation of the vaccination as a routine in India is still controversial. Here we have tried to critically analyse these issues in Indian context. However it is clear that cervical cancer vaccine is not an immediate panacea and cannot replace the cervical cancer screening which is mandatory in Indian context.

## 1. Introduction

Cervical Cancer is the third most common cancer in women worldwide with estimated 529000 new cases and 275000 deaths globally in 2008 [1]. More than 85% of the global burden of cervical cancer cases and 88% of cervical cancer deaths occur in developing countries. Indian contribution to cervical cancer cases is 25.4% and contribution to the mortality due to this disease is 26.5%. The age-standardized incidence and mortality rate of cervical cancer in India are 27.0 and 15.2, respectively [2]. The majority of the Indian women diagnosed with cervical cancer have never been screened for the disease and around 70% of these cases present in advance stages due to absence of any organized cervical cancer screening program. It has been estimated that there will be around 205496 new cases and 119097 deaths due to cervical carcinoma by 2020 in India [3]. The most important risk factor for development of cervical cancer is persistent infection by a high-risk subset of human papilloma virus (HPV) [4] which is the most common viral sexually transmitted infection. The lifetime risk of HPV infection for sexually active males and females is more than 50%. In sexually active women of less than 25 years of age prevalence of HPV is about 20% [5]. Most women's immune systems eliminate HPV infection spontaneously; however, for a very small proportion of women, the infection will persist and can cause precancerous changes in cells. HPV also causes anal cancer, with about 85 percent of all cases caused by HPV-16. HPV types 16 and 18 have also been found to cause close to half of vaginal, vulvar, and penile cancers [6].

The advent of HPV vaccination has advanced hopes that the dream of eradication of cervical cancer might be possible in future. Prophylactic vaccines for cervical cancer target HPV 16 and 18, the most common oncogenic types of HPV responsible for cervical cancer. Since cervical screening only detects precancerous and cancerous changes after they have occurred, HPV vaccination is primary prevention. As prophylactic HPV vaccination is not effective against all oncogenic HPV types, regular cervical screening is still necessary.

With this background, we are still in dilemma whether to implement the HPV vaccination in India as a routine or not? For any vaccination to be successful there are few salient points which must be considered:

1. basis of vaccination,
2. epidemiology of the disease,
3. vaccine efficacy,
4. cost effectiveness,
5. sociocultural impact,
6. impact of vaccination on screening.

## 2. Basis of Vaccination

The most important risk factor for development of cervical cancer is persistent infection by a high-risk subset of human papilloma virus (HPV) [4]. In a multicentric study in India, genotypes 16 and 18 alone or in coinfection with each other were detected in 76.3% cases and genotype 33 was the third most common type [7].

HPV are a large family of small double-stranded DNA viruses that has a specific predilection for squamous epithelium of skin and mucosa. The host immune response to natural infection with HPV is very slow and weak due to various reasons, that is, absence of viremia and virus induced necrosis, localized infection, lack of activation of Langhans cells, and inhibition of interferon synthesis and receptor signaling [8]. Thus only 50% of women infected with HPV develop antibodies and seroconversion may take 18 months. It is not necessary that these antibodies remain protective against reinfection with the same HPV type. The other associated risk factors for progression to high-grade dysplasia and cancer include persistence of HPV infection, infection with highly oncogenic HPV types, age more than 30 years, infection with multiple HPV types, and immunosuppression [9].

All the above factors led to development of prophylactic vaccines. At present, two prophylactic HPV vaccines, that is, the quadrivalent vaccine “Gardasil” and the bivalent vaccine “Cervarix” (Table 1), are available which have been prepared from purified L1 structural proteins by recombinant technology. These proteins self-assemble to form virus-like particles (VLPs) that induce a protective host immune response which is much stronger and long lasting and includes partial cross-protection against non-vaccine-related serotypes as compared to natural infection. The higher immune response to vaccine as compared to natural infection is attributable to high immunogenicity of VLPs, higher antigen dose in VLPs, and direct exposure of capsids to systemic immune responses [9]. HPV vaccines are designed for prophylactic use only; they do not clear existing HPV infection or treat HPV-related disease [10].

The quality of the antibody response is best for HPV 16 for both vaccines. The quality of the antibody response to HPV 6/11/18 for Gardasil is much poorer than its response to HPV 16 and higher dosages of HPV 11/16 were needed to prevent cross-inhibition by HPV 6/18, and as a result, the antigenic protein component of Gardasil that is necessary to affect an immunologic antibody response is high, at 120 *μ*g. Cervarix induces an equally high and sustained antibody response to HPV 16/18.

### 2.1. Adverse Effects

Side effects, such as autoimmune neurologic demyelination (paralysis, blindness, and death), albeit rare, have been associated with Gardasil due to higher antigenic protein load [11, 12]. Some have shown that young girls are more at risk than young boys for these neurologic side effects. Other common side effects such as pain and swelling at injection site, headache, fever, and vomiting are also reported. Safety of these vaccines when given in combination with other vaccines is not proven.

### 2.2. Target Population

The most appropriate target population for HPV vaccination will depend on the age at which individuals first get exposed to HPV. This depends largely on the sociocultural behaviour patterns of the region. In a survey among college students in Delhi, the age at sexual debut is earlier than the legal age at marriage, which is 18 years [13]. Thus in order to ensure that the recipients receive maximum protection, the target population should be young adolescents (9–13 years of age) who are supposed to be sexually inactive and mount a better immune response.

## 3. Epidemiology of Cervical Cancer in India

The incidence of cervical cancer and common HPV types along with the age-standardized mortality ratio has already been discussed but Mattheij et al. in their review questioned the epidemiology data of cancer cervix in India [14]. They analysed that the cancer registries and surveillance systems in India provide an inadequate basis for information because they are not complete or comprehensive in their coverage of every region in India. Cancer incidence data published by the main agencies, that is, National Cancer Registry Programme (NCRP) of India, the Cancer Atlas of India, the CI5, and the GLOBOCAN, has unequal representation of different parts of India, that is, under representation of east, far north, and rural India, as their data is mainly collected in the major cities, hospitals, and medical colleges.

It is well known that the efficacy of any intervention cannot be measured without monitoring or follow-up on epidemiological data. If the vaccine is to be implemented in a given country, every subpopulation should be represented equally. The World Health Organization (WHO) advises that the epidemiology of the disease should be known and be of sufficient importance to justify its prioritization and that surveillance systems should be capable of assessing the impact of a vaccine intervention following its introduction [15]. An effective surveillance system for HPV vaccine requires that the baseline incidence, prevalence, and mortality rates of cervical cancer are established. There is no general account in the literature of cancer surveillance in India.

The government of India had suspended the research on HPV vaccination by Programme for Appropriate Technology in Health (PATH), a USA-based not-for-profit nongovernmental organization (NGO) in 2010 due to the public concerns about its safety [16] which generated many controversies and reevaluation.

Cervical cancer may be a major cause of cancer in females, but cancer registries show that incidence rates are significantly declining (noted between years 1982 and 2005). This declining trend is also described in many studies [17]. Swaminathan et al. projected a 46% decrease in the incidence of cervical carcinoma in 2015 in Chennai [18]. Age-standardized cervical cancer rate per 100,0000 population of India (27) is lower as compared to other developing countries, that is, Zimbabwe (50.0) and Brazil (Goiania) (38) [19]. This data again raises the question of whether we need a vaccination or a comprehensive screening programme for cervical cancer as our first priority. Although both strategies can go side by side, the money constraint and the low GDP of India are the prohibitory factors for simultaneous implementation.

HPV vaccination programmes should only proceed where there are both strong epidemiological evidence and adequate surveillance and monitoring systems. In the absence of comprehensive cancer surveillance, World Health Organization criteria with respect to monitoring effectiveness of the vaccine and knowledge of disease trends cannot be fulfilled.

## 4. Efficacy and Duration of Protection

The aim of the HPV vaccines is to prevent cervical cancer but because of the long natural history of this disease it may well be a couple of decades before this difference can be documented. The decrease in the prevalence of cervical cancer may be the impact of effective screening programme rather than prevention by vaccine. Therefore, it has been agreed that efficacy can be measured by surrogate markers only, namely, the occurrence of new HPV infections and development of high-grade cervical intraepithelial neoplasia (CIN 2+) disease. In this respect, both the bivalent and quadrivalent HPV vaccines have demonstrated remarkable efficacy in phase II and phase III trials.

For questions regarding duration of protection, most models assumed that the vaccine provided either 10-year or lifetime protection. At this time, the minimum antibody titre level that confers protective efficacy has not been determined yet. For the quadrivalent HPV vaccine, titres specific to vaccine HPV types peaked at month 7 after the initial vaccine dose, declined through month 24, and stabilized at levels above baseline. Anti-HPV titters remained similar at month 60. For the bivalent HPV vaccine, antibody titres for both HPV 16 and HPV 18 peaked at month 7 after the initial dose and reached a plateau that was sustained from month 18 through month 76. A recent mathematical model of the immunological data from the bivalent HPV vaccine predicts that antibody titres above baseline may be observed 20 years after vaccination [20]. The actual effectiveness of HPV vaccination in the female population will also depend on levels of vaccine uptake or coverage and compliance in completing all vaccine doses.

The bivalent vaccine seems more efficacious against nonvaccine HPV types 31, 33, and 45 than the quadrivalent vaccine, but the differences were not all significant and might be attributable to differences in trial design. Efficacy against persistent infections with types 31 and 45 seemed to decrease in bivalent trials with increased follow-up, suggesting a waning of cross-protection; more data are needed to establish duration of cross-protection [21]. Cross-protection has also been reported against 31/33/45 and other high-risk HPV genotypes for both vaccines. These findings mean that Cervarix is 91% effective against HPV types that cause adenocarcinoma and 83% effective overall against squamous cell carcinoma. Compare that with Gardasil, which is 78% effective overall against HPV types that cause adenocarcinoma and 73% effective against HPV types that cause squamous cell carcinoma.

As it is very difficult to determine that in which person HPV infection will persist and lead to disease progression because most of the HPV infection resolve spontaneously, the usefulness of HPV vaccination is dubious. These arguments raise the value of HPV vaccination. Although it is clear that HPV vaccination cannot replace the cervical screening programmes as it does not protect against all HPV types, the vaccination at the right age could decrease the infection during peak ages of sexual activity and the screening can be delayed. Vaccination and the screening together may decrease the cervical cancer risk substantially.

## 5. Cost Efficacy

The direct cost of HPV vaccine is very high as compared to other vaccines used in immunization programme which prohibits its use in large percentage of females. Coverage of three doses of DPT and polio (the high priority vaccines) in extended programme for immunization in India was 60% as estimated by WHO-UNICEF and 90% by national estimates and for hepatitis B it was 8% by WHO estimates and 68% by national estimates in year 2005 [22]. This low coverage was surprising in the presence of enormous funding, good awareness among parents and health care workers, and various national health programmes. The other important point is that it will take decades for the cervical cancer rate to decline even if the vaccine is affordable and widely available [23]. Thus to invest the money in HPV vaccination in the absence of proper evidence on its safety, efficacy, and long term protection in developing countries like India seems to be inappropriate although the individual protection whenever affordable cannot be denied.

Public sector spending in health is very low in India (India spent 3.9% of its GDP on health in 2011 [24]), making it difficult for the government to independently take on the task of introducing the vaccine in the national immunization programme, without external support. Thus, although the vaccine is available for personal use in India, it has not been implemented at the population level.

## 6. Social Acceptance

There are certain sociocultural issues associated with the HPV vaccine because it targets a sexually transmitted infection (STI) and primarily targets female adolescents and young adults. These issues will significantly influence the willingness of health policy makers, health care providers, parents, and adolescent and young girls to receive vaccination. Out of these parental awareness and attitude towards the HPV vaccine are likely to be major determinants of acceptability.

## 7. Impact of Vaccination on Screening

HPV vaccination is expected to reduce the rate of abnormal Pap tests, and this will ultimately weaken the positive predictive value of cytology. It is also expected to reduce the need for common excisional treatments for cervical dysplasia in vaccinated women which is an important outcome of vaccination. Thus it appears that, once HPV vaccination is a routine, one can consider the HPV test as a primary screen, with triage to cytology in women who test HPV positive [25], and then the screening can be reduced by lengthening the screening interval and perhaps delaying the initial screening to 25 years of age. Women and physicians must understand the fact that a woman who chooses to be vaccinated may gain individual protection, but the overall rate of cervical cancer will not be affected. The most important thing is that women still need to be screened, even if they have been vaccinated [26].

## 8. Conclusion

Although the introduction of HPV vaccination is a scientific advancement in the cancer prevention, HPV vaccine is not an immediate panacea and optimization of its clinical use is still required. Cancer registration and surveillance systems should be extended across all population groups, including rural, northern, and eastern populations, and vital registry systems should be established for the collection of mortality data as the surveillance data is critical before any vaccine implementation in general population. Besides this we should concentrate on organized cervical cancer screening programme. Proven and cost effective methods such as VIA, VILI, PAP smear, and HPV DNA tests remain the most feasible prevention strategies in low resource countries of Indian subcontinent.

## Figures and Tables

**Table 1:** HPV vaccines.

HPV vaccine	Protection against HPV genotypes (amount)	Adjuvant	Vaccination age	Schedule	FDA approval
Gardasil	6, 11, 16, 18 (20/40/40/40 mcg)	Amorphous aluminium hydroxyl phosphate sulphate	Routine vaccination in girls of 11-12 years. Health provider's discretion between 9 and 11 years. Catch-up vaccination permitted till 26 years	0, 2, and 6 months	Approved
Cervarix	16, 18 (20/20 mcg)	Aluminum hydroxide and monophosphoryl lipid A		0, 1, and 6 months	

## References

[1] Mishra GA, Pimple SA, Shastri SS (2011). An overview of prevention and early detection of cervical cancers. *Indian Journal of Medical and Paediatric Oncology*.

[2] Saxena U, Sauvaget C, Sankaranarayanan R (2012). Evidence based Screening, early diagnosis and treatment strategy of cervical cancer for national policy in low resource countries: example of India. *Asian Pacific Journal of Cancer Prevention*.

[3] Ferlay J, Bray F, Pisani P, Parkin DM (2004). *GLOBOCAN, 2002 Cancer Incidence, Mortality and Prevalence Worldwide*.

[4] Walboomers JM, Jacobs MV, Manos MM, Bosch FX, Kummer JA, Shah KV (1999). Human papillomavirus is a necessary cause of invasive cervical cancer worldwide. *The Journal of Pathology*.

[5] Evander M, Edlund K, Gustafsson A (1995). Human papillomavirus infection is transient in young women: a population-based cohort study. *Journal of Infectious Diseases*.

[6] Muñoz N, Castellsagué X, de González AB, Gissmann L (2006). Chapter 1: HPV in the etiology of human cancer. *Vaccine*.

[7] Basu P, Roychowdhury S, Bafna UD (2009). Human papillomavirus genotype distribution in cervical cancer in India: results from a multi-center study. *Asian Pacific Journal of Cancer Prevention*.

[8] Mariani L, Venuti A (2010). HPV vaccine: an overview of immune response, clinical protection, and new approaches for the future. *Journal of Translational Medicine*.

[9] Forcier M, Musacchio N (2010). An overview of human papillomavirus infection for the dermatologist: disease, diagnosis, management, and prevention. *Dermatologic Therapy*.

[10] Ault KA (2007). Effect of prophylactic human papillomavirus L1 virus-like-particle vaccine on risk of cervical intraepithelial neoplasia grade 2, grade 3, and adenocarcinoma in situ: a combined analysis of four randomised clinical trials. *The Lancet*.

[11] DiMario FJ, Hajjar M, Ciesielski TA (2010). A 16-year-old girl with bilateral visual loss and left hemiparesis following an immunization against human papilloma virus. *Journal of Child Neurology*.

[12] Sutton I, Lahoria R, Tan IL, Clouston P, Barnett MH (2009). CNS demyelination and quadrivalent HPV vaccination. *Multiple Sclerosis*.

[13] Bhatla N, Joseph E (2009). Cervical cancer prevention & the role of human papillomavirus vaccines in India. *Indian Journal of Medical Research*.

[14] Mattheij I, Pollock AM, Brhlikova P (2012). Do cervical cancer data justify HPV vaccination in India? Epidemiological data sources and comprehensiveness. *Journal of the Royal Society of Medicine*.

[15] Vaccine Introduction Guidelines: adding a vaccine to a national immunization programme -decision and implementation. http://www.who.int/immunization/hpv/plan/vaccine_introduction_guidelines_who_2005.pdf.

[16] Lamontagne DS, Sherris JD (2013). Addressing questions about the HPV vaccine project in India. *The Lancet Oncology*.

[17] Dhillon PK, Yeole BB, Dikshit R, Kurkure AP, Bray F (2011). Trends in breast, ovarian and cervical cancer incidence in Mumbai, India over a 30-year period, 1976–2005: an age-period-cohort analysis. *British Journal of Cancer*.

[18] Swaminathan R, Shanta V, Ferlay J, Balasubramanian S, Bray F, Sankaranarayanan R (2011). Trends in cancer incidence in Chennai city (1982–2006) and statewide predictions of future burden in Tamil Nadu (2007–16). *National Medical Journal of India*.

[19] Cancer incidence in five continents [Internet]. http://ci5.iarc.fr/.

[20] Armstrong EP (2010). Prophylaxis of cervical cancer and related cervical disease: a review of the cost-effectiveness of vaccination against oncogenic HPV types. *Journal of Managed Care Pharmacy*.

[21] Malagon T, Drolet M, Boily MC (2012). Cross-protective efficacy of two human papillomavirus vaccines: a systematic review and meta-analysis. *The Lancet Infectious Diseases*.

[22] Giftson Senapathy J, Umadevi P, Kannika PS (2011). The present scenario of cervical cancer control and HPV epidemiology in India: an outline. *Asian Pacific Journal of Cancer Prevention*.

[23] Schiffman M, Wacholder S (2009). From India to the world—a better way to prevent cervical cancer. *The New England Journal of Medicine*.

[24] World Health Organization http://www.who.int/countries/ind/en/.

[25] Cox JT (2010). Is the HPV test effective as the primary screen for cervical cancer? Examining the evidence. *OBG Management*.

[26] Wilyman J (2013). HPV vaccination programs have not been shown to be cost-effective in countries with comprehensive Pap screening and surgery. *Infectious Agents and Cancer*.

